# Pharmacovigilance on cannabidiol as an antiepileptic agent

**DOI:** 10.3389/fphar.2023.1091978

**Published:** 2023-02-10

**Authors:** Ilaria Ammendolia, Carmen Mannucci, Luigi Cardia, Gioacchino Calapai, Sebastiano Gangemi, Emanuela Esposito, Fabrizio Calapai

**Affiliations:** ^1^ Department of Clinical and Experimental Medicine, University of Messina, Messina, Italy; ^2^ Department of Biomedical and Dental Sciences and Morphological and Functional Imaging, University of Messina, Messina, Italy; ^3^ Department of Human Pathology of Adult and Childhood “Gaetano Barresi”, University of Messina, Messina, Italy; ^4^ Department of Chemical, Biological, Pharmaceutical, and Environmental Sciences, University of Messina, Messina, Italy

**Keywords:** cannabidiol, cannabis, adverse reactions, pharmacovigilance, epilepsy

## Abstract

**Introduction:** Cannabidiol (CBD) is an active chemical contained in the plant *Cannabis sativa*. It is a resorcinol-based compound that crosses the blood-brain barrier without causing euphoric effects. CBD has a plethora of pharmacological effects of therapeutic interest. CBD has been authorized in the European Union as an anticonvulsant against serious infantile epileptic syndromes, but its safety profile is still not sufficiently described.

**Methods:** With the goal of expanding information on the safety of CBD use as an antiepileptic agent beyond the most common side effects known through clinical studies, an analysis of serious case reports on suspected adverse reactions (SARs) to CBD licensed as an anti-epileptic drug found in the EudraVigilance database is reported in this article. EudraVigilance is a system purchased by the European Medicines Agency (EMA) for monitoring the safety of medicinal products marketed in Europe.

**Results:** The most frequent serious SARs to CBD in EudraVigilance were epilepsy aggravation, hepatic disorders, lack of efficacy, and somnolence.

**Discussion:** Based on our analysis, the following precautions should be adopted for appropriate monitoring of potential adverse effects, more attention towards possible CBD medical use as an antiepileptic: awareness of interactions with other drugs, epilepsy aggravation, and drug effectiveness.

## 1 Introduction

Cannabidiol (CBD) or 2-[(6R)-6-Isopropenyl- 3-methyl-2-cyclohexen-1-yl]-5-pentyl-1,3-benzenediol was first isolated from Cannabis sativa in 1940, and its structure was described by Mechoulam et al., in 1963. CBD is a meroterpenoid obtained by the alkylation of alkyl resorcinol with a monoterpene unit (Tura et al., 2019; Boulebd et al., 2022). Products containing CBD are generally ingested or inhaled and are used to treat epilepsy and generalized severe pain or anxiety experienced as part of conditions such as palliative care (Calapai et al., 2019; Taylor et al., 2019). The intense first-pass metabolism influences the absorption and bioavailability of CBD when administered orally (Franco et al., 2020). Once in the blood, CBD is highly bound to plasma proteins (97%), and its intake can cause co-administered drugs to be displaced from protein binding (Elmes et al., 2015). The metabolism of CBD involves several CYP450 enzymatic isoforms, including CYP2C19, CYP3A4, CYP1A2, CYP2B6, CYP2C8, CYP2C9, CYP2E1, CYP2J2, and CYP3A5/7 (Lucas et al., 2018), which can mediate hydroxylation reactions resulting in hydroxylated products that can be further oxidized to form dihydroxylated metabolites. Phase two reactions are catalyzed by 5′-diphosphoglucuronosyltransferase (UGT) isoforms that promote glucuronidation, making metabolites more easily eliminated (Brown and Winterstein, 2019). The long half-life of CBD is due to its slow release, redistribution from lipid-rich compartments, and significant enterohepatic circulation (Foster et al., 2019).

Knowledge of the mechanism of action of CBD is still not exhaustive, and its effects on the central nervous system are only partially mediated by the activation of the endocannabinoid system (Calapai et al., 2020). Several findings show that it also acts as an activator of nuclear factor erythroid 2-related factor 2 (Nrf2) (Atalay et al., 2021). Due to the low binding affinity of CBD, cannabinoid receptors 1 and 2 (CB1R and CB2R) are not primary targets for this substance. CBD acts as a negative allosteric modulator of CB1R, and it acts as a partial agonist at CB2R (Liou et al., 2008). Furthermore, it can increase anandamide concentration through the inhibition of fatty acid amide hydrolase (FAAH), its main degradative enzyme, and it inhibits its reuptake (Galaj and Xi, 2019). Several biological effects induced by CBD are mediated by agonist activity on the peroxisome proliferator-activated gamma receptor (PPARγ), competitive inhibition of adenosine uptake, adenosine A2A receptor antagonism, and activation of transient receptor potential cation channel subfamily V, members 1 and 2 (TRPV1,2). CBD is also a blocker of the equilibrative nucleoside transporter (ENT) and the orphan G-protein-coupled receptor GPR55, and it enhances the activity of the 5-HT1A receptor (Russo et al., 2005; Devinsky et al., 2014). Further evidence indicates a role for CBD as a modulator of *µ*- and *δ*-opioid receptors (Kathmann et al., 2006), in addition to mitochondrial processes that may be relevant in the treatment of mitochondria-related diseases (Chan and Duncan, 2021).

Currently, it is possible to prescribe galenic preparations containing raw cannabis material in Europe, the United Kingdom, Canada, and Australia. CBD is also sold as an herbal supplement in Europe and many other countries (Iftikhar et al., 2021). As an antiepileptic agent, a CBD-based drug licensed as Epidyolex has been approved. It contains 100 μg/mL of delta-9-tetrahydrocannabinol-free sesame oil. Its registration was also carried out thanks to the numerous preclinical and clinical data that were included in the dossiers prepared for market authorization (Arzimanoglou et al., 2020).

To our knowledge, no systematic post-marketing study has been published on potential adverse reactions linked to CBD use in epileptic patients. To contribute to the definition of the safety of CBD as an antiepileptic drug, this article analyzed real-world data on suspected adverse reactions (SARs) to CBD-licensed products for epilepsy collected from EudraVigilance, a database system containing SARs to drugs yet licensed for the market or being currently studied in clinical trials in the European Union (EU) (https://www.ema.europa.eu/en/human-regulatory/research development/pharmacovigilance/eudravigilance). The European Medicines Agency (EMA) is responsible for overseeing of EudraVigilance, a system built to collect and analyze reports of suspected adverse effects from national medicines regulatory authorities and pharmaceutical companies in the European Economic Area (EEA). With the aim of expanding information on the safety of CBD use as an antiepileptic agent beyond the most common side effects (such as fever, diarrhea, and loss of appetite) generally emerging from clinical studies, in this article we presented a descriptive analysis of serious case reports on SARs to CBD products licensed as an antiepileptic drug found in the EudraVigilance database.

## 2 Methodology

The EudraVigilance database is directed and controlled by EMA on behalf of the EU. Individual Case Safety Reports (ICSRs) in this data bank describe SARs to CBD.

The EudraVigilance data system contains all ICSRs reported by a healthcare professional or a non-healthcare professional to a European Union national competent authority or a marketing authorization holder. Only ICSRs reporting SARs to CBD contained in licensed drugs as the suspected drug signaled by healthcare professionals were retrieved from 2019, when marketing authorization was granted by the EMA, to 22/10/2022. Licensed drugs containing CBD were authorized in 2019 by EMA for the following therapeutic indications: “use as adjunctive therapy of seizures associated with Lennox-Gastaut syndrome or Dravet syndrome, in conjunction with clobazam, for patients 2 years of age and older, as indicated in the Summary of Product Characteristics by EMA; use as adjunctive therapy of seizures associated with tuberous sclerosis complex for patients 2 years of age and older” (https://www.ema.europa.eu/en/documents/product-information/epidyolex-epar-product-information_en.pdf).

ICSRs are displayed as cases originated by EEA countries or not. In this article, reports include data coming from United Kingdom, because, despite Brexit in EudraVigilance data from this country are considered as part of EEA. The EEA area presently consists of 30 countries: Austria, Belgium, Bulgaria, Croatia, the Czech Republic, Cyprus, Denmark, Estonia, Finland, France, Germany, Greece, Hungary, Iceland, Ireland, Italy, Latvia, Liechtenstein, Lithuania, Luxembourg, Malta, the Netherlands, Norway, Poland, Portugal, Romania, Slovakia, Slovenia, Spain, and Sweden. However, the medical use of CBD is not legal in all these countries. A public version of the EudraVigilance database (http://www.adrreports.eu/en/search.html) has been used. EudraVigilance allows access to reports of suspected side effects/adverse drug reactions submitted electronically by national medicine regulatory authorities and pharmaceutical companies. In terms of data selection criteria, all ICSRs reporting SARs linked to the only CBD-based drug licensed in the EU were considered and selected based on the Medical Dictionary for Regulatory Activities (MedDRA) (MedDRA, 2022). SARs associated with CBD used for other purposes were excluded. MedDRA is a standardized, clinically validated international medical terminology employed by regulatory authorities and the biopharmaceutical industry. It is published by the International Council for Harmonization and is primarily used for coding cases of adverse effects in clinical study reports and pharmacovigilance databases and to facilitate searches in these databases (MedDra and pharmacovigilance, 2016). Information on patient characteristics (age group and sex), type of adverse reaction (often more than one for each ICSR), primary source qualification, and concomitant drugs were provided for all ICSRs. Only serious cases reported by healthcare professionals were considered for this analysis. According to the International Council on Harmonization E2D guidelines, ICSRs are classified as serious if they are life-threatening, result in death, cause or prolong hospitalization or disability, are determined as a congenital anomaly/birth defect, or are associated with another medically important condition. A descriptive statistical analysis was carried out in the present study. The data were analyzed using the statistical software SPSS version 28.0.0.0 (190).

## 3 Results

ICSRs reporting a licensed drug based on CBD as a suspected drug as a possible cause of SARs, during the period between August 2019 and October 2022 were retrieved from EudraVigilance (checked on 31/12/2022).

**FIGURE 1:**
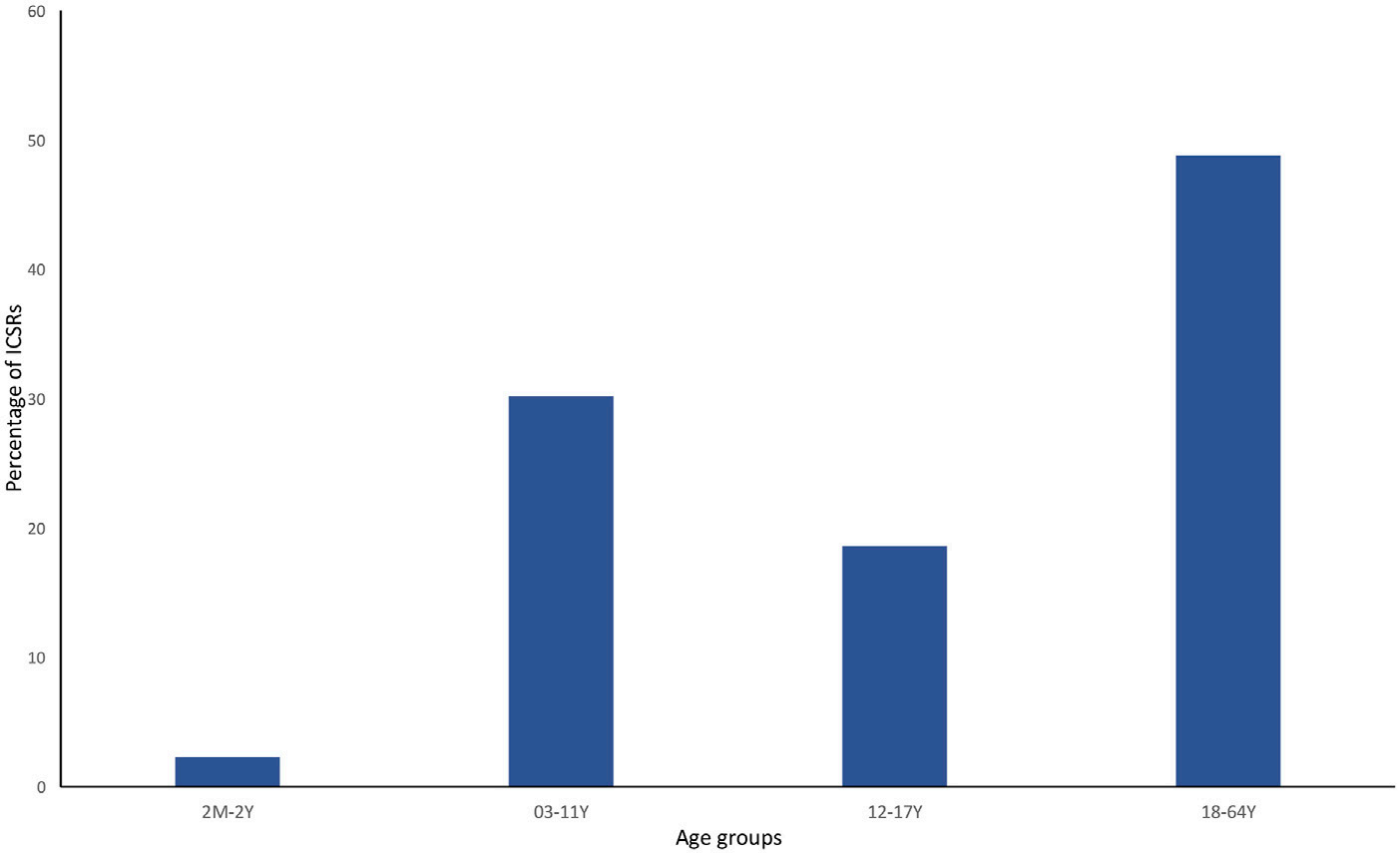
Percentage of serious individual case safety reports (ICSRs) (N = 44) in the EudraVigilance database related to cannabidiol use as an antiepileptic medication in the years 2019–2022, distributed according to age. 2 M–2 Y = 2 months–2 years; 03–11 Y = 3 years–11 years; 12–17 Y = 12–17 years; and 18–64 Y = 18–64 years.

Currently, the total number of ICSRs of SARs linked to CBD licensed as an antiepileptic drug traceable in EudraVigilance for the years 2019–2022 is 162 (as of 31/12/2022). In brackets, the number and percentage for each EEA country with respect to the total are reported. Most ICSRs have been reported by France (34.6%), followed by Germany (33.3%), Spain (11.1%), Italy (8.0%), the United Kingdom (8.0%), Norway (3.1%), Austria (2.5%). Serious ICSRs accounted for 29.0% (47/162) of all ICSRs associated with CBD use in epilepsy in the EEA (Table 1). All of the data included in ICSRs containing SARs for CBD licensed as an antiepileptic agent have been transferred to a new database and statistically analyzed. The distribution of age groups, calculated on serious 43 ICSRs (four ICSRs were missing for age indication) shows that adults aged 18–64 years (48.8%) are the most affected, followed by children aged 3–11 years (30.2%), adolescents aged 12–17 years (18.6%), and children aged 2 months to 2 years (2.3%) (Figure 1).

**TABLE 1:** Individual Case Safety Reports (ICSRs) with suspected adverse reactions (SARs) to cannabidiol (CBD) use as an antiepileptic medication in the European Economic Area (EEA) and United Kingdom in the years 2019–2022, distributed by country of origin and severity. European countries reporting fewer than four ICSRs are not reported.

*Country of origin*	Cases
France	56
Germany	54
Spain	18
Italy	13
United Kingdom	13
Norway	5
Austria	4
*Serious and non-serious ICSRs*	
Serious and non-serious ICSRs	162
Serious ICSRs and percentage of the total number of ICSRs	47 (29.0%)

Despite widespread knowledge that drug adverse reactions occur more frequently (nearly twice as frequently as men) in women (Salvo et al., 2013; Zucker and Prendergast, 2020), men have the highest number of serious ICSRs of SARs to licensed CBD compared to women (62.2% vs 37.8%) in EudraVigilance. SARs were more frequent in cases where CBD was taken in conjunction with other drugs (68.1%) than in cases where CBD was administered alone. Six of the 47 serious cases (or 12.8%) resulted in death. Table 2 displays the total number of non-serious SARs and the number of serious SARs listed by country of origin.

**TABLE 2:** Suspected adverse reactions (SARs) in the European Economic Area (EEA) and United Kingdom according to gender, concurrent use of cannabidiol with other drugs, and death, reported in serious individual case safety reports (ICSRs), with suspected adverse reactions (SARs) related to the prescription of cannabidiol as an antiepileptic drug in the years 2019–2022. The data are reported as a percentage of the total number of ICSRs (N = 47).

	%
Gender in serious ICSRs	37.8/62.2 (female patients/male patients)
Serious ICSRs with concurrent use of cannabidiol with other drugs	68.1
Reported deaths as an outcome of SARs in serious ICSRs	12.8

Table 2 also lists the adverse reactions more frequently reported in serious CBD-related ICSRs. The most common suspected SAR is “aggravation”, intended to mean a worsening of the existing epileptic disease. This is followed by reports of “hepatic disorders” (including elevated liver enzymes), CBD ineffectiveness, and severe somnolence (Table 3). In terms of severity distribution, serious cases were grouped as follows, according to EudraVigilance data: death 12.8%, hospitalization 59.6%, and not recovered 27.7%.

**TABLE 3:** More frequent suspected adverse reactions (SARs) reported in serious individual case safety reports (ICSRs) related to cannabidiol use as an antiepileptic medication in the years 2019–2022. The data are reported as SARs in single serious ICSRs (N = 47). More SARs can be reported in single ICSRs. SARs occurring only once are not reported.

Suspected adverse reactions to cannabidiol reported in serious ICSRs	Number of cases reported in ICSRs
Aggravation	12
Hepatic disorders and elevated liver enzyme values	9
Drug ineffectiveness	5
Somnolence	5
Emesis	3
Weight loss	2
Aggressive behavior	2
Anorexia	2
Urinary retention	2

**FIGURE 2:**
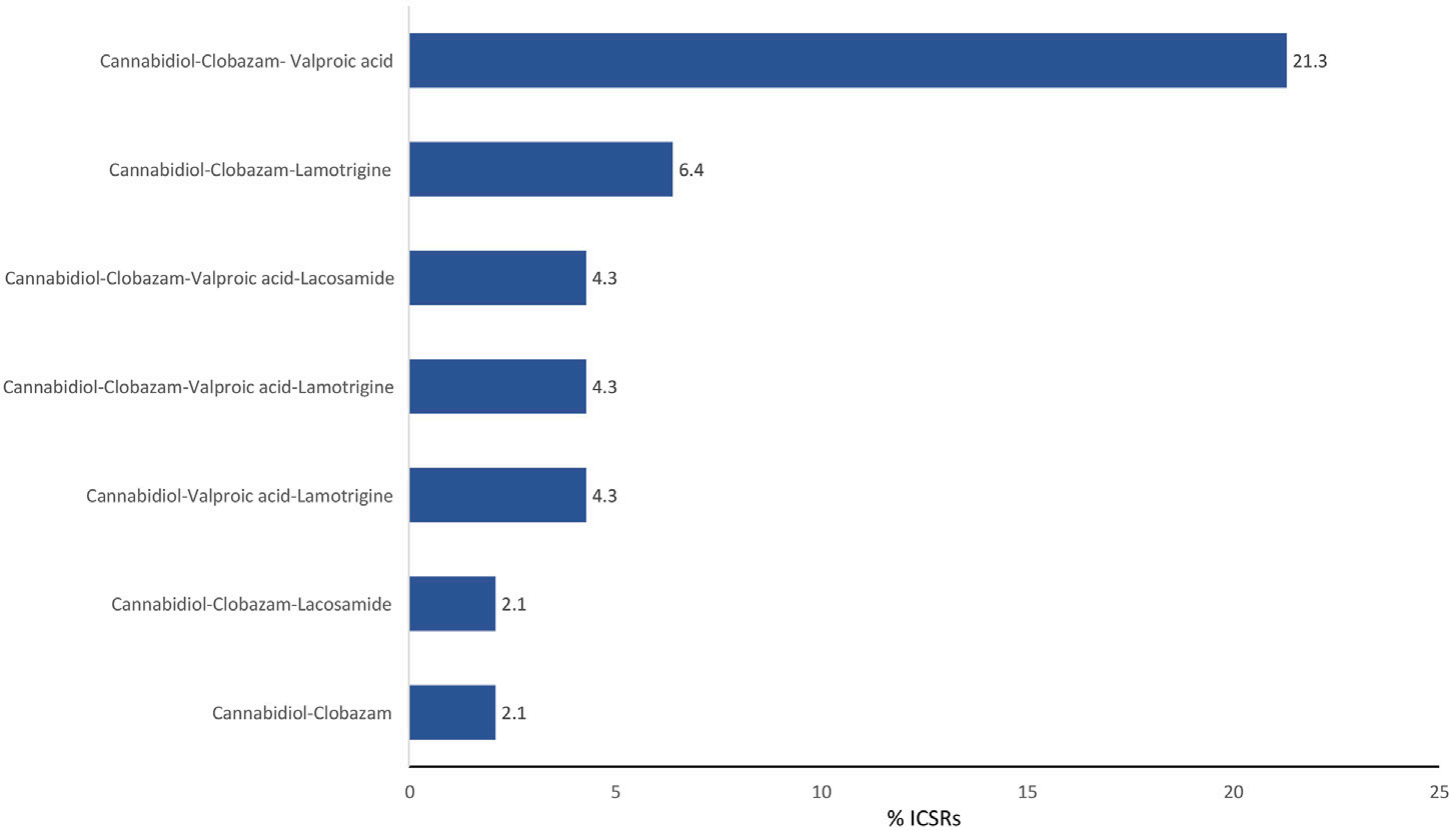
Combination of drugs prescribed with cannabidiol for epilepsy in the years 2019–2022 in serious cannabidiol-related individual case safety reports (ICSRs) in the EudraVigilance database. ICSRs = 47. The data are expressed as a percentage of the total number of serious ICSRs (N = 47).

The antiepileptics clobazam, valproic acid, lamotrigine, lacosamide, rufinamide, and levetiracetam are the drugs more commonly prescribed in serious ICSRs reporting SARs to licensed CBD used against epilepsy, in descending order (Table 4). The frequency of prescription of the four most prescribed drugs in association with CBD reported in serious ICSRs and related to SARs was analyzed. Only 2.1% of serious ICSRs with CBD were prescribed with clobazam, while CBD alone with valproic acid was prescribed in 4.3% of serious ICSRs (Figure 2). Clobazam and valproic acid prescriptions were found in 51.1% and 40.4% of serious CBD-related ICSRs, respectively (Table 4). Data show that the most common drug association in serious ICSRs reporting SARs to CBD is between CBD, clobazam, and valproic acid, followed by the association of CBD-clobazam-lamotrigine (Figure 2). Finally, the results show that the number of serious ICSRs increases as a result of the association of CBD with the other two drugs.

**TABLE 4:** Drugs more frequently prescribed in serious individual case safety reports (ICSRs) reporting suspected adverse reactions (SARs) related to cannabidiol (CBD) use as an antiepileptic drug in the years 2019–2022.

Drugs prescribed with CBD	Number of serious ICSRs reporting suspected adverse reactions to CBD	Prescription with CBD (% of total number of serious ICSRs; N = 47)
Clobazam	24	51.1
Valproic acid	19	40.4
Lamotrigine	7	14.9
Lacosamide	6	12.8
Rufinamide	5	10.6
Levetiracetam	4	8.5

## 4 Discussion

The EMA designated CBD, with the commercial name Epidyolex, as an “orphan drug” (a medicine used in rare diseases) for the following indications: Dravet syndrome, 15/10/2014; Lennox-Gastaut syndrome, 20/03/2017; Tuberous sclerosis, 17/01/2018. Currently, safety information on CBD use as an antiepileptic is primarily based on data obtained from clinical studies that were analyzed for market authorization.

In general, clinical trial results indicate that CBD is a safe compound that causes only rare adverse reactions; however, concerns about safety have been raised, which are supported by experiments on laboratory animals. Nevertheless, the importance of this uncertainty in the clinical use of CBD has not been clearly defined (Ewing et al., 2019). CBD can produce direct adverse effects, as it has been observed in children treated for seizures resistant to other drugs. Some of these negative effects include elevated temperatures but also convulsions (Devinsky et al., 2019). These symptoms, together with temporary diarrhea and a loss of appetite, were confirmed by other studies, but the long-term effects of CBD are not definitively known (Laux et al., 2019; Wu et al., 2022).

The present work displays pharmacovigilance data from the EudraVigilance database. They are real-world data derived from pharmacovigilance signals about the use of CBD as an antiepileptic in the EU.

Descriptive data analysis indicates that in the context of the antiepileptic use of CBD, serious ICSRs are prevalent in male patients, when CBD is taken with other concomitant drugs, and in the age groups of adults 18–64 years (48.8%), followed by children aged 3–11 years (30.2%). The most common serious adverse reactions reported in ICSRs are “aggravation of epilepsy” and “hepatic disorders,” followed by “lack of effectiveness” and “somnolence.” Furthermore, 12.8% of cases result in death.

Multiple aspects of CBD pharmacology are associated with drug interactions potentially linked to its use with other medications, in particular with those able to influence CYP450 isoenzyme activity (Manikandan et al., 2018; Foster et al., 2019). In fact, CBD is an inhibitor of CYP450 enzymes such as CYP3A4 and CYP2C19 (Beers et al., 2021). To a lesser extent, CBD also has an inhibitory effect on the activity of CYP1A1, CYP1A2, CYP1B1, CYP2A6, CYP2B6, CYP3A5, CYP2D6, CYP2C9, and on the glucuronidation enzymes UGT1A9 and UGT2B7 (Qian et al., 2019). In this regard, it is important to note that in some cases, when high doses of CBD were used to treat childhood epilepsy, it was necessary to reduce the dose of co-administered synthetic antiepileptics. In particular, it has been observed that increasing the CBD dose reduces seric clobazam concentration and, at the same time, produces a 4–5–fold increase in its major active metabolite, N-clobazam (Karaźniewicz-Łada et al., 2021). Moreover, the enzymes CYP2C19 and CYP3A4 catalyze the formation of the metabolite 7-Hydroxy-CBD (7-OH-CBD) from CBD. This is the primary active metabolite that is equipotent with CBD and is directly conjugated by UDP-glucuronosyltransferases (Stout and Cimino, 2014). As previously stated, the occurrence of adverse reactions to CBD can also be attributed to drug-drug interactions, which are generally caused by the mechanism of CYP enzyme induction or inhibition (Patsalos et al., 2020).

The frequency of combinations of three drugs most commonly prescribed to those with serious CBD-related conditions indicates that the most common association involved in SARs is the prescription of CBD with clobazam and valproic acid, followed by the prescription of CBD with clobazam and lamotrigine.

EMA approved CBD for use as adjunctive therapy for seizures in conjunction with CLB; however, the combination of CBD with other drugs is common in epilepsy therapy. In this regard, it is worth noting that CBD was responsible for 31.9% of serious cases of SARs when used as monotherapy, which is an off-label use because CBD has only been authorized as an add-on therapy with clobazam for epilepsy. On this basis, the use of CBD in conjunction with valproic acid should also be considered an off-label prescription. However, the prescription of valproic acid with CBD for epilepsy seems to be frequent. Its use is most likely based on the safety profile of this combination emerging from clinical studies published after EMA approval (Patel et al., 2021; Scheffer et al., 2021). The majority of serious problems associated with CBD treatment can be attributed to interactions that this molecule may have when co-administered in polytherapy. The analysis of EudraVigilance data suggests that more attention be paid because, even though previous clinical studies showed a safer profile, the safety of this association in the real world has not been confirmed. Therefore, in patients receiving concurrent CBD/valproic acid therapy, the dose of these drugs may need to be adjusted.

Frequent aggravation of pre-existing conditions and lack of efficacy have yet to be reported in clinical studies with CBD, and in any case, they have not received the attention they deserve, while somnolence and potential hepatic impairment have already been detected together with potential drug-drug interactions (Devinsky et al., 2017; Devinsky et al., 2018). For a better understanding of the origins of these observed effects, it is possible to hypothesize that CBD’s multi-target pharmacological properties, such as stimulation of serotonergic and dopaminergic neuron firing, could explain them (French et al., 1997; De Gregorio et al., 2019). CBD has been shown to have a partial agonistic action on dopamine D2-like receptors, an action that may block the D2 receptors when they are overreactive or activate them when they are underactive (Shrader et al., 2020).

According to EudraVigilance data, “aggravation of epilepsy” is the most frequent SAR observed among serious ICSRs related to CBD. Epilepsy aggravation is a cause for concern because it increases the chance of physical injury and is also a risk factor for sudden unexpected death in epilepsy (Devinsky, 2011). Seizure aggravation has been mainly attributed to an “inverse pharmacodynamic effect,” in which specific effects of the drug on its antiseizure target lead to seizure worsening rather than improvement (Rogawski, 2020). Evidence from preclinical studies strongly indicates that CBD’s effects on seizure reduction involve neuronal excitability modulation *via* different mechanisms, making it difficult to explain how it could cause an inverse pharmacodynamic effect (Zavala-Tecuapetla et al., 2022). According to the Summary of Product Characteristics of CBD released by EMA as an antiepileptic drug, an increase in seizure frequency, as with other anticonvulsants, may occur during treatment with CBD, even though in phase three clinical trials, the frequency of status epilepticus was similar between the CBD and placebo groups (Epidyolex, 2022) (https://www.ema.europa.eu/documents/product-information/epidyolex-epar-product-information_en.pdf).

It has recently been suggested that using CBD in epilepsy without clobazam may result in reduced efficacy and seizure aggravation (Rogawski, 2020). Clobazam, a 1,5-benzodiazepine, is a positive allosteric modulator of synaptic GABA_A_ receptors that are widely used to treat focal and generalized seizures, and it is part of the treatment for epileptic encephalopathies such as Dravet syndrome and Lennox-Gastaut syndrome (Leahy et al., 2011; NG et al., 2011). Concerning aggravation, it should also be noted that several currently available antiepileptic drugs are contraindicated in Dravet syndrome and Lennox-Gastaut syndrome because they may cause a worsening of seizures. Dravet syndrome is a chronic pediatric epilepsy syndrome that begins in early childhood. Patients affected by Dravet syndrome generally have medically refractory epilepsy, and polytherapy is often required. First-line agents include valproate and clobazam, while sodium channel-blocking anticonvulsants such as carbamazepine and lamotrigine (the latter prescribed in some CBD-related ICSRs) are generally contraindicated in this syndrome (Wallace et al., 2016). Lennox-Gastaut syndrome is a severe epileptic and developmental encephalopathy. Several currently available antiepileptic drugs are contraindicated in Lennox-Gastaut syndrome because they may cause a worsening of seizures (Genton, 2000), including carbamazepine, oxcarbazepine, and pregabalin, which have been detected occasionally in single serious CBD-related ICSRs in the EudraVigilance database.

In our analysis of EudraVigilance data, no significant quantitative difference was found between the frequency of seizure aggravation and reduced efficacy observed in ICSRs where CBD was prescribed without CLB and ICSRs where both drugs were prescribed. However, our discovery of a high prevalence of ICSRs associated with epilepsy aggravation signaled by CBD use in clinical practice seems to be relevant because this issue had not previously emerged through clinical studies.

Death was reported as SAR in six cases, accounting for 12.8% of the total number of serious ICSRS to CBD in EudraVigilance. Death, particularly Sudden unexpected death in epilepsy (SUDEP), seems to be the leading cause of premature death in people affected by epilepsy, especially in those patients with refractory epilepsy, and its exact etiology is not known. However, research in animal models revealed that multiple neurotransmitters, including serotonin (5-HT) and adenosine, both of which are targets of CBD biological activity, may be involved in the pathophysiological mechanisms of SUDEP (Zhao et al., 2022).

Anyway, in general, CBD itself is a compound characterized by a low-risk profile, even when higher doses are prescribed (Chesney et al., 2020). Concerns about CBD medical use, on the other hand, may be related to potential interactions with the concurrent use of other drugs. This seems to be caused by CBD’s enzymatic inhibitory activity, particularly when high doses are used in conjunction with antiepileptic drugs, resulting in lower blood concentrations of these drugs (Gilmartin et al., 2021). Another aspect that needs to be further investigated is the possible CBD liver toxicity and its occurrence in clinical practice (Ewing et al., 2019).

The limitations of this pharmacovigilance study are represented by the restriction of data available in the public EudraVigilance database, and by the typical problems of the spontaneous reporting system, which are mainly related to under-reporting and potential information inaccuracies. However, knowledge can be obtained through the analysis of very large sample size data, which provides information on treatment practices in specific populations that are usually excluded from randomized clinical trials, in addition to the appropriateness of the prescription.

Finally, despite the fact that they are limited to the European area, real-world data from EudraVigilance indicate that serious adverse effects of CBD are prevalently affecting adults and children. Based on the data and concepts shown and expressed in this article, it is possible to assert that the clinical use of CBD as an antiepileptic agent requires more caution than previously applied. Precautions should be taken regarding the adoption of CBD in clinical practice, with greater awareness of its use with other drugs, avoiding off-label use when possible, appropriate surveillance of potential adverse effects, increased knowledge and attention to the possible aggravation of epilepsy, and the occurrence of potential and known CBD drug interactions.

## Data Availability

Publicly available datasets were analyzed in this study. This data can be found here: EUDRAVIGILANCE (https://www.adrreports.eu/en/search.html#).
